# Canadian Guidelines for Forensic Psychiatry Assessments and Report Writing—Executive Summary

**DOI:** 10.1177/07067437231200843

**Published:** 2023-09-28

**Authors:** Lisa A. L. Ramshaw, Sumeeta Chatterjee, Todd Tomita, Graham D. Glancy, Treena D. Wilkie

**Affiliations:** 1 Division of Forensic Psychiatry, Department of Psychiatry, Faculty of Medicine, University of Toronto, Centre for Addiction & Mental Health ( 7978 CAMH), Toronto, Canada; 2 Division of Forensic Psychiatry, Department of Psychiatry, Faculty of Medicine, University of Toronto, Forensic Service, 7978 Centre for Addiction & Mental Health (CAMH), Toronto, Canada; 3 12358 University of British Columbia, Toronto, Canada; 4 Division of Forensic Psychiatry, Department of Psychiatry, 12366 Faculty of Medicine, University of Toronto, Centre for Addiction & Mental Health (CAMH), Toronto, Canada; 5 Division of Forensic Psychiatry, Department of Psychiatry, Faculty of Medicine, University of Toronto, Centre for Addiction & Mental Health (CAMH), Toronto, Canada

**Keywords:** clinical practice guidelines, Forensic psychiatry, assessment, medicolegal issues

The Royal College of Physicians and Surgeons of Canada formally recognized forensic psychiatry as a subspecialty of psychiatry in 2011 (Royal College website). The Royal College has since accredited eight forensic psychiatry training programs across Canada, and each of these programs has implemented competence by design teaching and evaluation^1^ to ensure rigorous and consistent standards of training. Recognizing the importance of national standards, the *Canadian Guidelines for Forensic Psychiatry Assessments and Report Writing* were developed as a collaborative project. A five-member steering committee* wrote the initial guidelines. A ten-member national working group**, selected based upon expertise, regional representation, interest, and commitment, reviewed, and contributed to each guideline. This was followed by expert peer reviews, and then approval by the Canadian Academy of Psychiatry (CAPL) Board. The guidelines are intended for use as a national resource for trainees in the field, as well as being a reference for those conducting third-party assessments.

Forensic psychiatry third-party assessments involve criminal and civil matters. The first of ten *Canadian Guidelines for Forensic Psychiatry Assessments and Report Writing* has been published on the CAPL website.^2^ Representing each of the major areas of the field, the guidelines begin with the General Principles, which are provided as a link to all of the other guidelines. The specific guidelines include Fitness to Stand Trial, Criminal Responsibility, Violence Risk Assessments, Dangerous Offender and Long-Term Offender Assessments, and Sexual Behaviour and Sexual Offending Risk Assessments; and the four civil specific guidelines: Disability, Fitness for Work, Personal Injury, and Professional Misconduct and Malpractice (each with a general introduction to civil psychiatry link)**.**

All of the *Canadian Guidelines for Forensic Psychiatry Assessments and Report Writing* follow the same format. This includes an introduction to the topic with case law and legislation, a section on assessment, and a section on report writing. They are intended as a review of legal and psychiatric principles and to offer practical guidance in the performance of forensic evaluations, taking into account regional and legislative differences across the country. The guidelines do not address expert testimony, and with the exception of the Sexual Behaviour and Sex Offending Risk Assessment guideline and some potential recommendations, they do not address treatment.

The General Principles guideline provides an overview of the ethical considerations in forensic psychiatry, based on the CAPL Ethical Guidelines.^3^ This includes the necessity of conducting fair, objective, nonpartisan, and nonbiased assessments within the assessor's area of expertise, the importance of maintaining the highest level of professionalism, and the complexity of dual roles in forensic psychiatry. Other general areas include communication with third parties, recognizing that the forensic assessor's ultimate opinion will be objective and independent of the retaining party. The guidelines stress the importance of establishing the focus and the limitations of any assessment. Given the incentives implicit in the outcome of third-party assessments, the assessor must carefully weigh the reliability of the evaluee and other sources of information when forming opinions.

The assessment section of the General Principles guideline provides an approach to forensic evaluation including the interview and information-gathering process. Safety and privacy considerations that are fundamental during the evaluation are reviewed. The principles of informed consent are addressed and involve a review of the reason for assessment, the role of the assessor, and the limits to confidentiality. The assessment involves a detailed history and a sociocultural understanding of the evaluee. It is foundational to obtain and integrate multiple sources of information beyond the direct interview. This may include a review of the file information, collateral interviews, and adjunctive testing. Collateral interviews can include any source with first-hand knowledge of the evaluee.

The last section of the General Principles guideline provides an overview of the report structure. This includes the main headings of a forensic psychiatric report (including reason for assessment, sources of information, limits of confidentiality, background information, and opinions and recommendations). The importance of using descriptive language rather than medical jargon is also highlighted.

Each of the nine specific guidelines provides overviews of forensic psychiatry topics including the relevant case law and legislation, and areas of importance for each type of assessment and report. Examples of report templates are also provided.

These guidelines provide a practical resource for trainees and assessors and are intended to reflect current best practices within the field.^3–5^ The guidelines should not be construed as dictating one standard for forensic evaluations. Although they are intended to inform practice, they do not present all currently acceptable ways of performing such evaluations, and following these guidelines does not lead to a guaranteed outcome. The guidelines will be updated every 5 years, in consideration of evolving best practices, new case law and legislation, and user feedback.

*Steering Committee: Lisa Ramshaw, Treena Wilkie, Sumeeta Chatterjee, Todd Tomita, Graham Glancy.

**National Working Group: Todd Tomita, Alberto Choy, Mansfield Mela, Jeff Waldman, Richard Schneider, Brad Booth, Jocelyne Brault, Mathieu Dufour, Aileen Brunet, Lisa Ramshaw
