# Supplementary material for: Canadian Guidelines for Forensic Psychiatry Assessments and Report Writing—Executive Summary
Source: Can J Psychiatry. 2023 Sep 28;69(3):165–8. [Article in French] doi: 10.1177/07067437231200843 (PMC10874598; doi:10.1177/07067437231200843)
Supplement: Related texts

# Review article: Guide canadien sur l’expertise et la rédaction de rapports en psychiatrie légale : résumé

**Authors:** Lisa A. L. Ramshaw, Sumeeta Chatterjee, Todd Tomita, Graham D. Glancy, Treena D. Wilkie

**Affiliations:** 1 Professeure adjointe, division de psychiatrie légale, département de psychiatrie, faculté de médecine, Université de Toronto. Psychiatre, service de psychiatrie légale, Centre de toxicomanie et de santé mentale (CAMH), Toronto (Ontario).; 2 Professeure adjointe et directrice du programme de surspécialité en psychiatrie légale, division de psychiatrie légale, département de psychiatrie, faculté de médecine, Université de Toronto. Responsable et psychiatre légiste, service de psychiatrie légale, Centre de toxicomanie et de santé mentale (CAMH), Toronto (Ontario).; 3 Professeur adjoint de clinique, Université de la Colombie-Britannique.; 4 Professeure titulaire et directeur du programme de psychiatrie légale, division de psychiatrie légale, département de psychiatrie, faculté de médecine, Université de Toronto. Service de psychiatrie légale, Centre de toxicomanie et de santé mentale (CAMH), Toronto (Ontario).; 5 Professeure adjointe, division de psychiatrie légale, département de psychiatrie, faculté de médecine, Université de Toronto. Psychiatre légiste, médecin-chef suppléant, affaires et pratique médicales, chef du programme de soins complexes et de rétablissement pour les services médico-légaux, Centre de toxicomanie et de santé mentale (CAMH), Toronto (Ontario).

En 2011, le Collège royal des médecins et chirurgiens du Canada (CRMCC) a officiellement reconnu la psychiatrie légale comme une surspécialité de la psychiatrie (site Web du Collège royal). Depuis, le Collège royal a agréé huit programmes de formation en psychiatrie légale au Canada, et chacun de ces programmes a mis en œuvre une formation et une évaluation selon la compétence par conception (1) afin de veiller à ce que les normes de formation soient rigoureuses et cohérentes. Reconnaissant l’importance de se doter de normes nationales, le *Guide canadien sur l’expertise et la rédaction de rapports en psychiatrie légale* a été le fruit d’une collaboration. Un comité directeur composé de cinq membres* a rédigé les lignes directrices initiales. Un groupe de travail national constitué de dix membres**, sélectionnés en fonction de leur expertise, de leur représentation régionale, de leur intérêt et de leur implication, ont examiné chaque guide et y ont contribué. Cette étape a été suivie d’un examen par des pairs experts, puis de l’approbation du conseil d’administration de l’Académie canadienne de psychiatrie et droit (ACPD). Les guides sont destinés à servir de ressource nationale pour les apprenants du domaine de la psychiatrie légale, ainsi qu’à servir de référence pour les personnes qui effectuent des évaluations à la demande d’une tierce partie.

Les évaluations à la demande d’une tierce partie en psychiatrie légale concernent les affaires pénales et civiles. Le premier de la série de 10 *Guides sur l’expertise et la rédaction de rapports en psychiatrie légale* a été publié sur le site Web de l’ACPD (2). Représentant chacun des principaux domaines de la psychiatrie légale, les guides commencent par des principes généraux, qui servent de fondement pour tous les autres guides. Les thématiques abordées dans le cadre de la série sont l’aptitude à subir un procès, la responsabilité pénale, l’évaluation du risque de violence, l’évaluation des délinquants dangereux et des délinquants à contrôler, et l’évaluation du comportement sexuel et du risque de délinquance sexuelle; en ce qui concerne les procédures civiles, les thématiques abordées sont l’invalidité, l’aptitude au travail, les lésions corporelles, et l’inconduite et la faute professionnelle (chaque guide comportant une introduction générale reliée à la psychiatrie légale civile)**.**

Chaque *Guide canadien sur l’expertise et la rédaction de rapports en psychiatrie légal*e de la série a le même format, à savoir une introduction du sujet avec la jurisprudence et la législation, une section sur l’évaluation et une section sur la rédaction du rapport. Les guides ont pour but de passer en revue les principes juridiques et psychiatriques et d’offrir des conseils pratiques sur la réalisation d’évaluations médico-légales, en tenant compte des différences régionales et législatives qui existent au Canada. Les guides ne traitent pas des témoignages d’experts et, à l’exception de celui sur l’évaluation du comportement sexuel et du risque de délinquance sexuelle, et de certaines recommandations possibles, ils ne traitent pas du traitement.

Le guide présentant les principes généraux donne un aperçu des considérations éthiques en psychiatrie légale, en se fondant sur les directives éthiques de l’ACPD (3). Ces directives sont notamment la nécessité de mener des évaluations justes, objectives, non partisanes et non biaisées dans le domaine d’expertise de l’évaluateur, l’importance de maintenir le plus haut niveau de professionnalisme et la complexité des doubles rôles en psychiatrie légale. Parmi les autres domaines généraux, citons la communication avec les tierces parties, sachant que l’opinion finale de l’expert médico-légal sera objective et indépendante de la partie qui demande l’évaluation. Les guides soulignent l’importance de définir l’objectif et les limites de toute évaluation. Du fait des motivations implicites liées aux résultats des évaluations faites à la demande d’une tierce partie, l’évaluateur doit soigneusement peser la fiabilité de la personne évaluée et des autres sources d’information lorsqu’il se forme une opinion.

La section du guide portant sur les principes généraux consacrée à l’évaluation fournit une approche de l’évaluation médico-légale, comprenant l’entretien et le processus de recueil d’information. Les considérations relatives à la sécurité et à la confidentialité qui sont fondamentales pendant l’évaluation sont passées en revue. Les principes du consentement éclairé sont abordés et comportent un examen du motif de l’évaluation, du rôle de l’évaluateur et des limites de la confidentialité. L’évaluation requiert un historique détaillé et une compréhension socioculturelle de la personne évaluée. Il est fondamental d’obtenir et d’intégrer de multiples sources d’information au-delà de l’entretien direct. Il peut s’agir d’un examen des informations contenues dans le dossier, d’entretiens avec les proches et de tests complémentaires. Les personnes rencontrées dans le cadre de ces entrevues peuvent inclure n’importe quelle source ayant une connaissance directe de la personne évaluée.

La dernière section du guide portant sur les principes généraux donne un aperçu de la structure du rapport. Cela comprend les principales rubriques d’un rapport en psychiatrie légale (y compris le motif de l’évaluation, les sources d’information, les limites de la confidentialité, les renseignements généraux, les opinions et les recommandations). L’importance d’utiliser un langage descriptif plutôt qu’un jargon médical est également soulignée.

Chacun des neuf guides fournit une vue d’ensemble des aspects de la psychiatrie légale, y compris la jurisprudence et la législation pertinentes, ainsi que les domaines d’importance pour chaque type d’évaluation et de rapport. Des exemples de modèles de rapport sont également fournis.

Ces guides constituent une ressource pratique pour les apprenants et les évaluateurs, et sont censés mettre en évidence les meilleures pratiques actuelles dans le domaine (3,4,5). Les lignes directrices décrites dans les guides ne doivent pas être interprétées comme étant une norme à suivre en matière d’évaluations médico-légales. Bien qu’il ait pour but d’orienter la pratique, il ne présente pas toutes les manières actuellement acceptables de réaliser de telles évaluations et le fait de suivre les lignes directrices qui y figurent ne conduit pas à un résultat garanti. Les guides de pratique clinique seront mis à jour tous les cinq ans, en tenant compte de l’évolution des pratiques exemplaires, de la nouvelle jurisprudence et de la nouvelle législation, ainsi que des commentaires émis par ceux ayant recours aux guides.

*Comité directeur : Lisa Ramshaw, Treena Wilkie, Sumeeta Chatterjee, Todd Tomita, Graham Glancy.

**Groupe de travail national : Todd Tomita, Alberto Choy, Mansfield Mela, Jeff Waldman, Richard Schneider, Brad Booth, Jocelyne Brault, Mathieu Dufour, Aileen Brunet, Lisa Ramshaw

## References

[1] BoothBD ChatterjeeS , Watts J, et coll. *Toward a new model of training in Canadian forensic psychiatry*. J Am Acad Psychiatry Law. 2021;49(3):381-395.34016668 10.29158/JAAPL.200112-20

[2] RamshawL WilkieT ChatterjeeS , et coll. Guide canadien sur l’expertise et la rédaction de rapports en psychiatrie légal : Principes généraux *.* Académie canadienne de psychiatrie et droit; www. capl-acpd.org 2022 : 1-17. Accessible à l’adresse https://www.capl-acpd.org/wp-content/uploads/2022/06/01-Guidelines-FIN-FR-Web.pdf

[3] Académie canadienne de psychiatrie et droit. *Guide éthique à l’intention des médecins psychiatres légistes canadiens* . Ottawa (ON) : ACPD; 2019.

[4] BloomH SchneiderRD , dir. *Law and mental disorder – A comprehensive and practical approach* . Toronto (ON) : Irwin Law; 2013.

[5] GlancyG RegehrC. *Canadian landmark cases in forensic medical health* . Toronto (ON) : University of Toronto Press; 2020.
